# The STING pathway drives noninflammatory neurodegeneration in NGLY1 deficiency

**DOI:** 10.1084/jem.20242296

**Published:** 2025-07-11

**Authors:** Kun Yang, Gustavo Torres-Ramirez, Nicole Dobbs, Jie Han, Makoto Asahina, Reiko Fujinawa, Kun Song, Yun Liu, Weichun Lin, Angelica Oviedo, Chuo Chen, Lei Zhu, William F. Mueller, Kevin Lee, Tadashi Suzuki, Nan Yan

**Affiliations:** 1 Department of Immunology, University of Texas Southwestern Medical Center, Dallas, TX, USA; 2 Glycometabolic Biochemistry Laboratory, RIKEN Pioneering Research Institute, Saitama, Japan; 3 Takeda-CiRA Joint Program (T-CiRA), Kanagawa, Japan; 4 Department of Neuroscience, University of Texas Southwestern Medical Center, Dallas, TX, USA; 5 Pathology and Laboratory Medicine, Burrell College of Osteopathic Medicine, Las Cruces, NM, USA; 6 Department of Biochemistry, University of Texas Southwestern Medical Center, Dallas, TX, USA; 7 Grace Science, LLC, Menlo Park, CA, USA; 8 Grace Science Foundation, Menlo Park, CA, USA

## Abstract

The STING pathway is increasingly recognized as a key regulator of neuroinflammation in neurodegenerative disease, but its role in noninflammatory conditions remains unclear. We generated a postnatal inducible whole-body *Ngly1* knockout mouse (i*Ngly1*^−/−^) to model NGLY1 deficiency, an early-onset neurodegenerative disorder. i*Ngly1*^−/−^ mice exhibit progressive motor deficits, Purkinje cell loss, and shortened lifespan without evidence of gliosis or immune activation. Cell type–specific deletion of *Ngly1* in Purkinje cells or microglia failed to induce disease, suggesting multiple cell-intrinsic and cell-extrinsic signals are required. Genetic ablation of *Sting1* in i*Ngly1*^−/−^ mice rescues Purkinje cell loss, improves motor function, and extends lifespan. Single-nucleus RNA sequencing reveals proteostasis disruption in Purkinje cells, altered cerebellar granule cell subpopulations, and STING-dependent suppression of cholesterol biosynthesis in glia. Pharmacological inhibition of STING with an orally bioactive antagonist, VS-X4, significantly mitigates neuropathology and motor disease. These findings identify STING as a key mediator of neuropathology in NGLY1 deficiency and implicate a role of STING in noninflammatory neurological disease.

## Teaser

NGLY1 deficiency is an early-onset neurodegenerative disorder with no effective therapy. Here, we describe an inducible *Ngly1* knockout mouse model, i*Ngly1*^−/−^, that reveals pronounced loss of Purkinje cells in the cerebellum without neuroinflammation. *Sting1 *knockout or STING inhibitor VS-X4 significantly rescues i*Ngly1*^−/−^ disease in mice.

## Introduction

The STING pathway is a key component of the innate immune system, and plays a crucial role in response to infections, as well as autoinflammatory and autoimmune diseases (Decout et al., 2021). Recent studies further implicate STING in neurological disease, and pharmacological inhibition of STING is emerging as an attractive therapeutic strategy to treat neurodegeneration (Yang et al., 2024). In the brain, STING can be activated by the abnormal presence of mitochondrial or genomic DNA, which triggers the cytosolic DNA sensor cGAS to produce cGAMP, leading to STING activation (Chu et al., 2021; Sliter et al., 2018; Udeochu et al., 2023; Xie et al., 2023; Yu et al., 2020). Activation of STING signaling is associated with both sporadic age-related and familial early-onset neurodegenerative conditions.

N-glycanase 1 (NGLY1) is an evolutionarily conserved cytosolic deglycosylating enzyme, which is involved in the quality control of misfolded N-glycoproteins in eukaryotes (Suzuki et al., 2016). Mutations in the *NGLY1* gene in humans have been associated with a rare early-onset inherited disorder known as NGLY1 deficiency (also referred to as *NGLY1*-related congenital disorder of deglycosylation, NGLY1-CDDG, OMIM#615273). Affected individuals typically present with global developmental delay, neurological dysfunction (including movement disorder, hypotonia), elevated liver transaminases, and hypolacrima/alacrima (Enns et al., 2014). Currently, there is no treatment available for NGLY1 deficiency. In recent years, significant progress has been made in understanding the mechanisms underlying this disorder (Suzuki and Fujihira, 2024). We and others have observed mitochondrial dysfunction in both *Ngly1*^−/−^ mouse cells and cells derived from patients with NGLY1 deficiency (Han et al., 2020; Kong et al., 2018; Yang et al., 2018). We further elucidated that impaired mitophagy in *NGLY1*-deficient cells leads to the activation of the mtDNA-cGAS-STING pathway. However, the role of STING activation in the neuropathogenesis of NGLY1 deficiency has not yet been explored in vivo.

In this study, we generated a postnatal inducible whole-body *Ngly1* knockout mouse model that develops progressive neurological disease. Conditional *Ngly1* knockout and bulk and single-nucleus RNA sequencing (snRNA-seq) analysis of brain tissues revealed a disease mechanism that involves multiple neuronal cell types, leading to eventual Purkinje neuron loss in the cerebellum. We also present genetic and pharmacological evidence supporting STING as a key mediator of noninflammatory neuropathology associated with NGLY1 deficiency.

## Results

### Characterization of postnatal inducible whole-body *Ngly1* knockout mice

**Figure 1.**
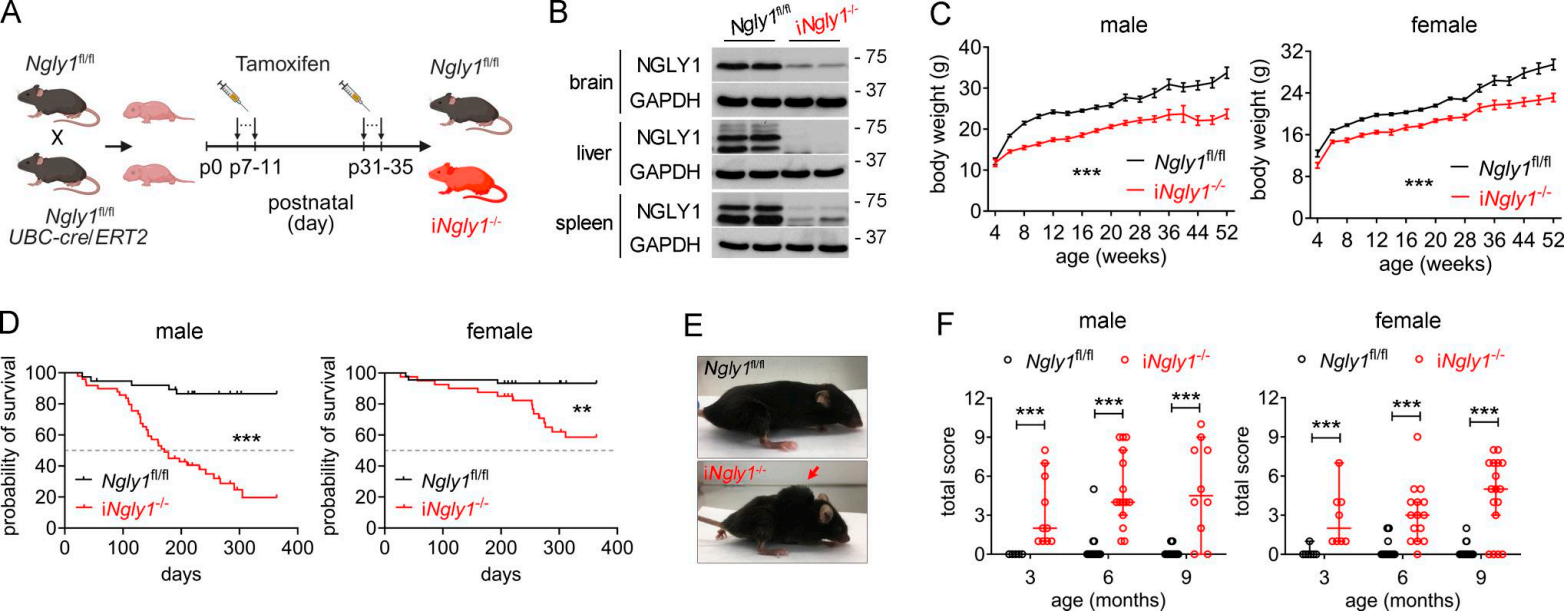
**Characterization of postnatal inducible whole-body *Ngly1* knockout mice. (A)** Schematic diagram showing the generation of postnatal inducible whole-body *Ngly1* knockout (i*Ngly1*^−/−^) mice. **(B)** Western blot analysis of the NGLY1 protein in brain, liver, and spleen of i*Ngly1*^−/−^ and *Ngly1*^fl/fl^ control mice. The data were verified in at least two independent experiments. **(C)** Body weight of i*Ngly1*^−/−^ and *Ngly1*^fl/fl^ mice (*n* = 4–25 each time point). Data are shown as the mean ± SEM. Two-way ANOVA. ***P < 0.001. **(D)** Survival curves of i*Ngly1*^−/−^ (*n* = 49 males, 40 females) and *Ngly1*^fl/fl^ (*n* = 38 males, 45 females) mice. Log-rank (Mantel–Cox) test. **P < 0.01; ***P < 0.001. **(E)** Representative image of kyphosis of i*Ngly1*^−/−^ mice at 3 mo old. **(F)** Neurological deficit score of i*Ngly1*^−/−^ and *Ngly1*^fl/fl^ mice. Data are shown as median ± 95% CI. Mann–Whitney *U* test. ***P < 0.001. Source data are available for this figure: SourceData F1.

Germline whole-body knockout of *Ngly1* in C57BL/6 genetic background is embryonically lethal (Fujihira et al., 2017; Yang et al., 2018). To circumvent this, we generated *Ngly1*^fl/fl^*UBC-cre/ERT2* mice, enabling postnatal inducible knockout of *Ngly1* via tamoxifen injection (referred to as i*Ngly1*^−/−^; Fig. 1 A). Knockout of NGLY1 in i*Ngly1*^−/−^ mice was confirmed at the protein level in clinically relevant tissues, including brain, liver, and spleen (Fig. 1 B). We established a large cohort of i*Ngly1*^−/−^ mice and monitored their disease phenotypes over the course of 1 year. Compared with their *Ngly1*^fl/fl^ littermate controls, i*Ngly1*^−/−^ mice exhibited reduced body weight (Fig. 1 C) and a significantly shortened lifespan (Fig. 1 D). Notably, i*Ngly1*^−/−^ mice began to display striking motor abnormalities, such as tremors and poor coordination/ataxia, as early as 3 mo of age (Video 1). Visible kyphosis also developed in i*Ngly1*^−/−^ mice at 3 mo and progressively worsened with age (Fig. 1 E). Using a composite 12-point scoring system with four different assays for mouse motor and coordination (hindlimb clasping, ledge walking, gait, kyphosis) (Guyenet et al., 2010), we observed notable motor defects in i*Ngly1*^−/−^ mice by 3 mo of age, with phenotypes worsening over time (Fig. 1 F). Therefore, the i*Ngly1*^−/−^ mouse model recapitulated key neurological symptoms reported in patients with NGLY1 deficiency.

**Video 1.** **Motor defect of i*Ngly1*** ^**−/−**^ **mice.**

### Purkinje cell loss in both i*Ngly1*^−/−^ mouse and NGLY1 deficiency patient cerebellum

**Figure S1.**
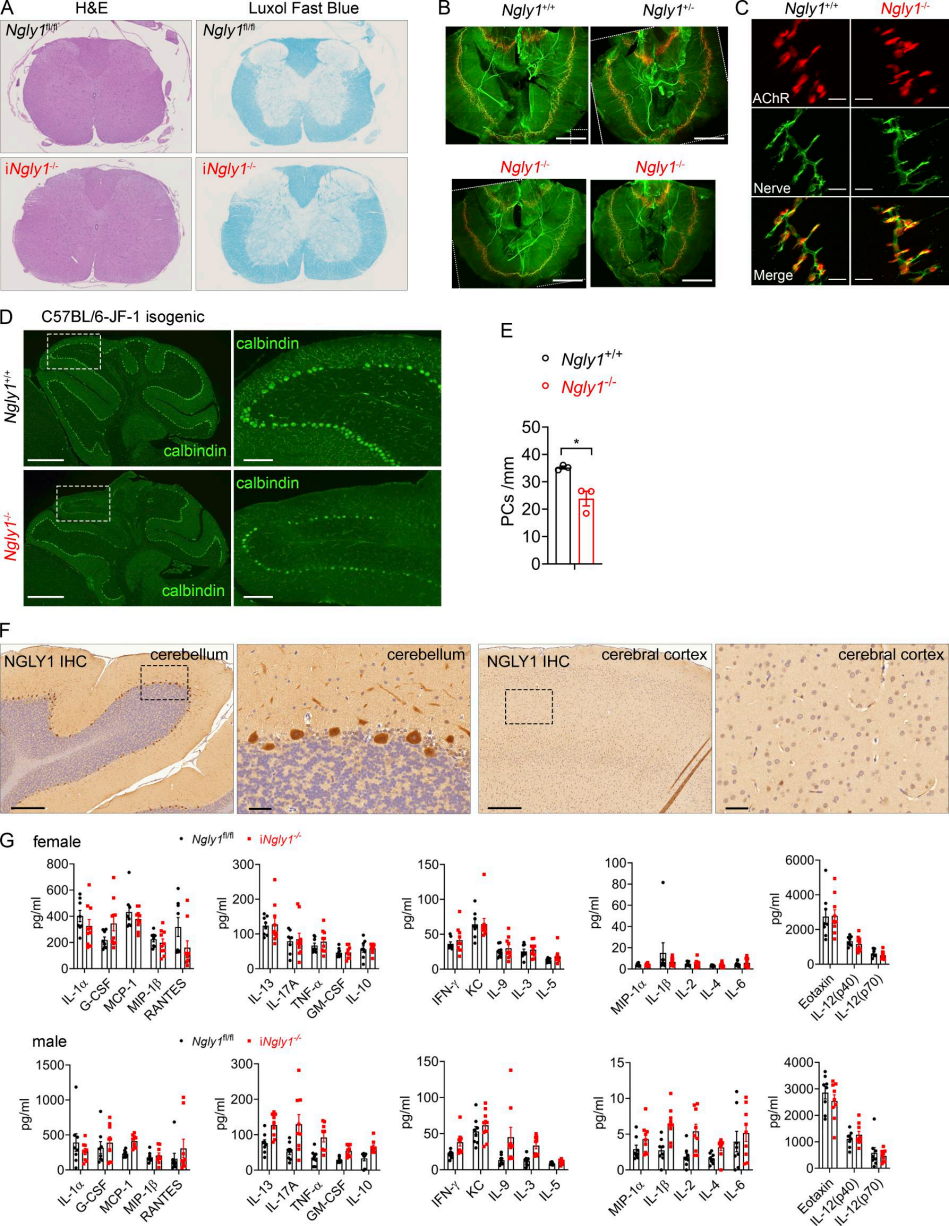
**Characterization of *Ngly1*-deficient mice. (A)** H&E and Luxol fast blue analysis of the thoracic spinal cord of 6-mo-old i*Ngly1*^−/−^ and i*Ngly1*^fl/fl^ control mice. The data are representative of at least three independent mice per genotype. **(B and C)** Innervation (B) and synaptic size (C) of E18.5 *Ngly1*^−/−^ and littermate fetus (*n* = 2 per genotype). α-Bungarotoxin was used to label postsynaptic ACh receptors in the muscle (red), and antibody against syntaxin was used to label presynaptic nerves (green). Low-power (10×) views of the phrenic nerve/diaphragms (B); scale bar, 2 mm. Note: the images were rotated after acquisition for presentation to maintain consistent anatomical orientation across samples. Nontissue triangular regions in the corners were outlined with dashed white lines. High-power (63×) views of neuromuscular junctions in the diaphragm (C); scale bar, 20 μm. Presynaptic nerve terminal, green; postsynaptic AChRs, red. **(D)** Representative fluorescent IHC staining of Purkinje cell marker calbindin (green) in cerebella of 5-wk-old JF1/BL6 isogenic F1 *Ngly1*^−/−^ and *Ngly1*^+/+^ control mice. *n* = 3 per genotype. Scale bar, 500 μm (left), 100 μm (right). **(E)** Quantification of calbindin-immunoreactive Purkinje cells shown in D. Data are shown as the mean ± SEM. Student’s *t* test. *P < 0.05. **(F)** Representative IHC staining of NGLY1 in cerebellum (left) and cerebral cortex (right) of cynomolgus macaque monkeys. Scale bar, 500 μm (zoom-out), 100 μm (zoom-in). **(G)** Multiplex cytokine analysis of female and male i*Ngly1*^−/−^ (*n* = 10 females, 9 males) and *Ngly1*^fl/fl^ (*n* = 8 females, 8 males) mouse serum.

Given that neurological diseases are the primary clinical manifestations in NGLY1 deficiency patients, we next investigated the underlying neuropathology in i*Ngly1*^−/−^ mice. Histopathology analysis of the spinal cord in i*Ngly1*^−/−^ mice revealed no outstanding abnormalities in H&E staining or demyelination in Luxol fast blue staining (Fig. S1 A). We also explored the possibility of neuromuscular junction abnormalities by examining phrenic nerve and diaphragms of E18.5 C57BL/6 *Ngly1*^−/−^ embryos. The innervation pattern and individual neuromuscular junction formation in *Ngly1*^−/−^ embryos were comparable to those in *Ngly1*^+/+^ control (Fig. S1 B and C), ruling out a role of NGLY1 in neuromuscular junction.

**Figure 2.**
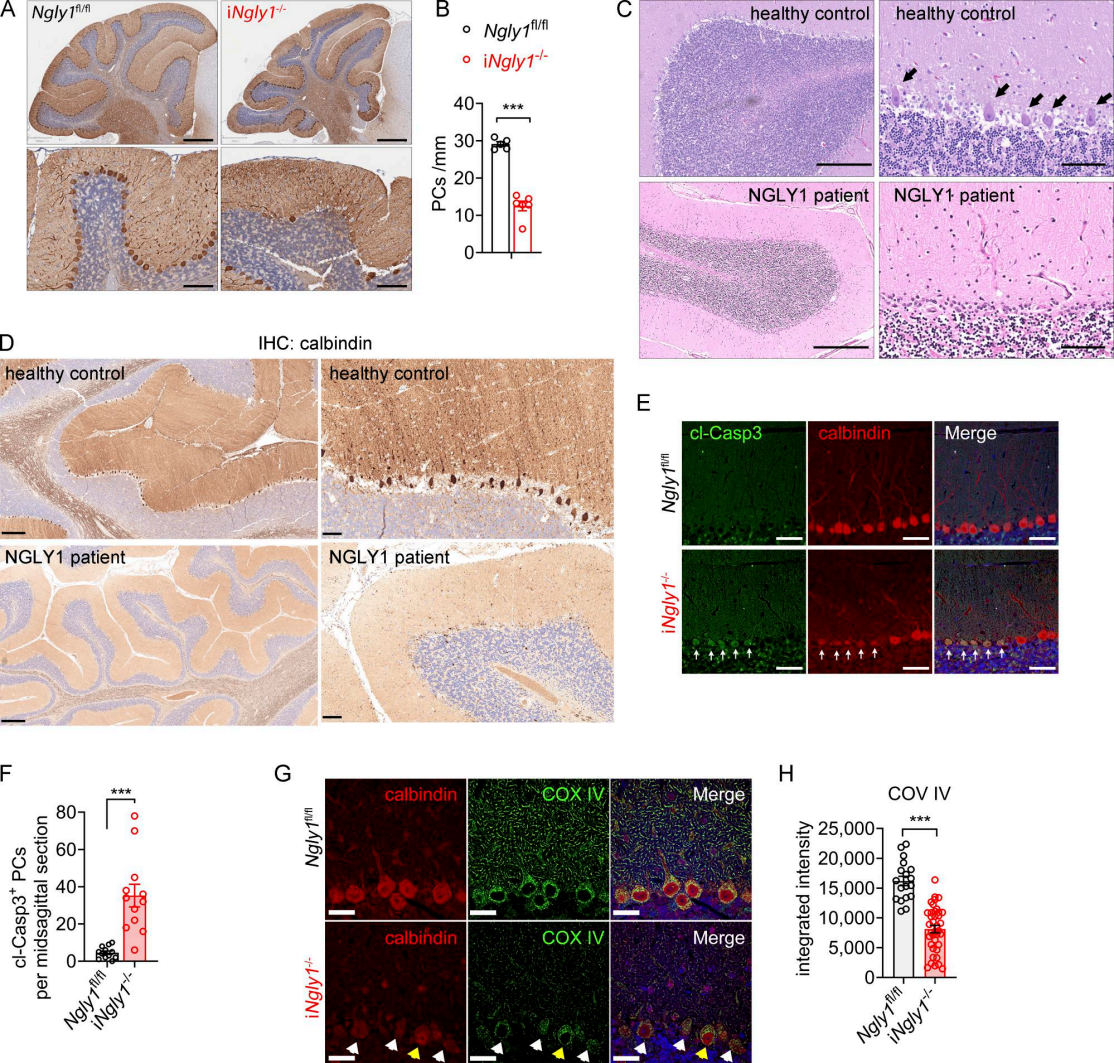
**Purkinje cell loss in i*Ngly1*** ^**−/−**^ **mice and an NGLY1 patient. (A and B)** IHC staining of calbindin in cerebella of 6-mo-old i*Ngly1*^−/−^ and *Ngly1*^fl/fl^ mice. Scale bar, 500 μm (upper), 100 μm (lower). Quantification of calbindin-immunoreactive Purkinje cells is shown in B. Data are shown as the mean ± SEM. Unpaired Student’s *t* test. ***P < 0.001. **(C and D)** Representative images of H&E staining (C) and calbindin IHC-DAB staining (D) of cerebella of a NGLY1 deficiency patient and an age-matched healthy individual. Scale bar, 500 μm (left), 100 μm (right). Note: cerebellar atrophy in NGLY1 deficiency patient (low magnification in D). **(E and F)** Fluorescent IHC staining of cleaved caspase-3 (cl-Casp3, green) and calbindin (red) in cerebella of 6-wk-old i*Ngly1*^−/−^ and *Ngly1*^fl/fl^ mice. Scale bar, 50 μm. Quantification of cl-Casp3^+^ Purkinje cells per midsagittal section is shown in F. Data are the mean ± SEM of i*Ngly1*^−/−^ (*n* = 12) and *Ngly1*^fl/fl^ (*n* = 12) mice. Unpaired Student’s *t* test. ***P < 0.001. **(G and H)** Fluorescent IHC staining of mitochondrial marker COX IV (green) and calbindin (red) in cerebella of 6-wk-old i*Ngly1*^−/−^ and i*Ngly1*^fl/fl^ control mice. Scale bar, 25 μm. Quantification of COX IV fluorescence intensity in Purkinje cell soma is shown in H. Data are the mean ± SEM of at least 18 Purkinje cells of i*Ngly1*^−/−^ (*n* = 6) and *Ngly1*^fl/fl^ (*n* = 4) mice. Unpaired Student’s *t* test. ***P < 0.001.

We next examined the brain pathology of i*Ngly1*^−/−^ mice. While H&E staining revealed no discernible difference between i*Ngly1*^−/−^ and *Ngly1*^fl/fl^ control brains (data not shown), immunohistochemical (IHC) staining of Purkinje cell marker calbindin revealed remarkable loss of Purkinje cells in the cerebellum of i*Ngly1*^−/−^ mice (Fig. 2, A and B). To corroborate this key neuropathological finding, we examined Purkinje cells in C57BL/6-JF1 isogenic *Ngly1*^−/−^ mice, a viable germline *Ngly1* knockout model on mixed genetic background (Asahina et al., 2021). Similar to i*Ngly1*^−/−^ mice, C57BL/6-JF1 isogenic *Ngly1*^−/−^ mice also exhibited reduced numbers of Purkinje cells compared with isogenic wild-type controls (Fig. S1, D and E). Furthermore, we analyzed the autopsied cerebellum of a 5-year-old NGLY1 deficiency patient with a history of pediatric neurodegeneration (Stuut et al., 2021). Compared with an age-matched healthy individual, the NGLY1 patient’s cerebellum exhibited extensive Purkinje cell loss and atrophy as evidenced in H&E staining and IHC staining of calbindin (Fig. 2, C and D). These findings from two *Ngly1*^−/−^ mouse models and an NGLY1 deficiency patient underscore a critical role of NGLY1 in the survival of Purkinje cells. Using brain tissue from a nonhuman primate cynomolgus macaque, we observed widespread expression of the NGLY1 protein in neuronal cells, with the highest levels in Purkinje cells (Fig. S1 F). This may explain the high susceptibility of Purkinje cells to NGLY1 loss.

To gain insight into Purkinje cell loss in i*Ngly1*^−/−^ mouse cerebellum, we stained cerebellar tissue from 6-wk-old i*Ngly1*^−/−^ mice (prior to the onset of motor symptoms) with the apoptotic marker cleaved caspase-3. Purkinje cells exhibiting abnormal morphology and diminishing calbindin staining in these young i*Ngly1*^−/−^ mouse cerebellum were also stained positive for cleaved caspase-3, indicating they were undergoing apoptosis (Fig. 2, E and F). Previous in vitro studies using both mouse- and patient-derived cells have shown that *Ngly1*-deficient cells display abnormal mitochondria and impaired mitochondrial respiration (Kong et al., 2018; Panneman et al., 2020; Yang et al., 2018). To explore this further in vivo, we examined the mitochondrial marker COX IV in i*Ngly1*^−/−^ mouse cerebellum. We found a marked reduction in COX IV staining in both soma and dendrites of i*Ngly1*^−/−^ Purkinje cells, suggesting impaired mitochondrial function (Fig. 2, G and H).

### *Ngly1* deficiency does not lead to overt inflammation in the cerebellum

**Figure 3.**
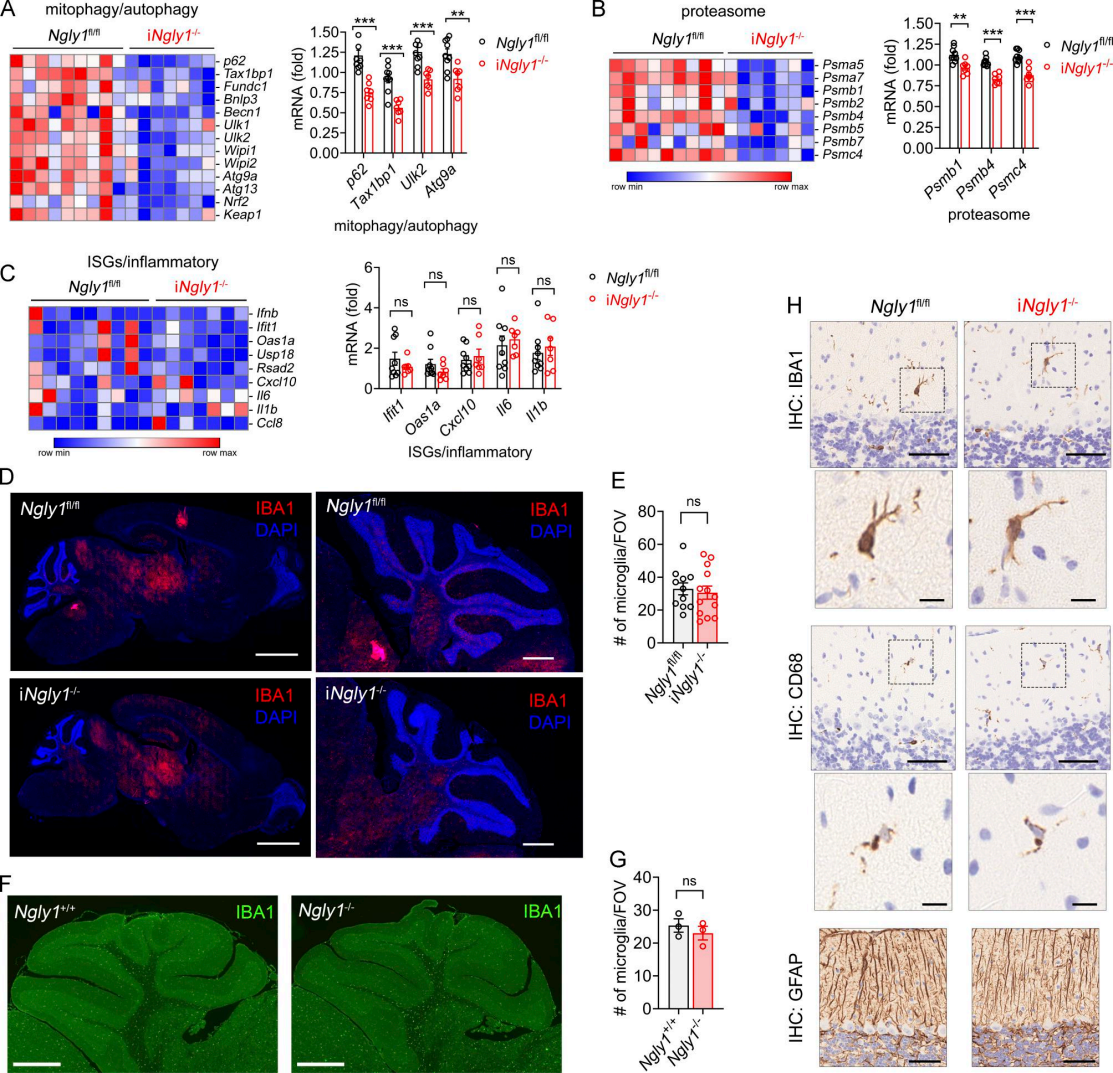
**Lack of neuroinflammation in i*Ngly1*** ^**−/−**^ **mice. (A–C)** Heatmap of mRNA expression in cerebella of i*Ngly1*^−/−^ (*n* = 7) and i*Ngly1*^fl/fl^ control (*n* = 9) mice. mRNA was measured by RT-qPCR array. Bar graphs are shown as the mean ± SEM. Unpaired Student’s *t* test. **P < 0.01; ***P < 0.001; ns, not significant. **(D)** Representative fluorescent IHC staining of microglial marker IBA1 (red) in cerebella of 3-mo-old i*Ngly1*^−/−^ and i*Ngly1*^fl/fl^ control mice. The nucleus was stained with DAPI (blue). Scale bar, 2 mm (left), 500 μm (right). Images were acquired using Zeiss AxioScan with automated stitching. **(E)** Quantification of microglia numbers (IBA1, red in D) in the FOV of the cerebellar molecular layer of i*Ngly1*^−/−^ (*n* = 13) and *Ngly1*^fl/fl^ (*n* = 11) mice. Data are the mean ± SEM. Unpaired Student’s *t* test, ns, not significant. **(F)** Representative fluorescent IHC staining of microglial marker IBA1 (green) in cerebella of 5-wk-old C57BL/6-JF1 isogenic F1 *Ngly1*^−/−^ and *Ngly1*^+/+^ control mice. Scale bar, 500 μm. Images were acquired using Hamamatsu NanoZoomer with automated stitching. **(G)** Quantification of microglial numbers (IBA1, green in F) in the FOV of the cerebellar molecular layer of C57BL/6-JF1 isogenic F1 *Ngly1*^−/−^ (*n* = 3) and *Ngly1*^+/+^ (*n* = 3) mice. Data are the mean ± SEM. Unpaired Student’s *t* test, ns, not significant. **(H)** Representative IHC staining of microglial marker IBA1, activation marker CD68, and astrocyte marker GFAP in cerebella of 6-mo-old i*Ngly1*^−/−^ and i*Ngly1*^fl/fl^ control mice. The data were verified in at least three mice. Scale bar, 50 μm (IBA1 upper, CD68 upper, and GFAP), 10 μm (IBA1 lower, CD68 lower). FOV, field of view.

Bulk RNA analysis of i*Ngly1*^−/−^ cerebellar tissue revealed a global reduction in autophagy- and mitophagy-related genes, as well as proteasome subunit genes, consistent with previously defined NGLY1 function (Yang et al., 2018) (Fig. 3, A and B). However, we were surprised to find no difference in IFN-stimulated genes (ISGs) or inflammatory genes between i*Ngly1*^−/−^ and control mouse cerebella (Fig. 3 C). Additionally, we did not observe any change in IBA1 staining, which would suggest microgliosis or neuroinflammation, in the cerebellum of either i*Ngly1*^−/−^ (C57BL/6) or germline *Ngly1*^−/−^ (C57BL/6-JF1) cerebellum (Fig. 3, D–G). We further performed 3,3′-diaminobenzidine (DAB) IHC staining for CD68, IBA1, and GFAP in *iNgly1*^−/−^ brains at 6 mo of age, when substantial Purkinje cell loss was evident. We did not observe a notable increase in the expression of glial activation marker CD68 in the *iNgly1*^−/−^ cerebellum (Fig. 3 H). Additionally, the morphology of microglia, astrocytes, and Bergmann glia in *iNgly1*^−/−^ appeared indistinguishable from that in control mice (Fig. 3 H). In addition, a multiplex cytokine array analysis of i*Ngly1*^−/−^ mouse serum showed no signs of systemic inflammation (Fig. S1 G). These findings suggest that *Ngly1* deficiency causes noninflammatory neurodegeneration in the cerebellum.

**Figure S2.**
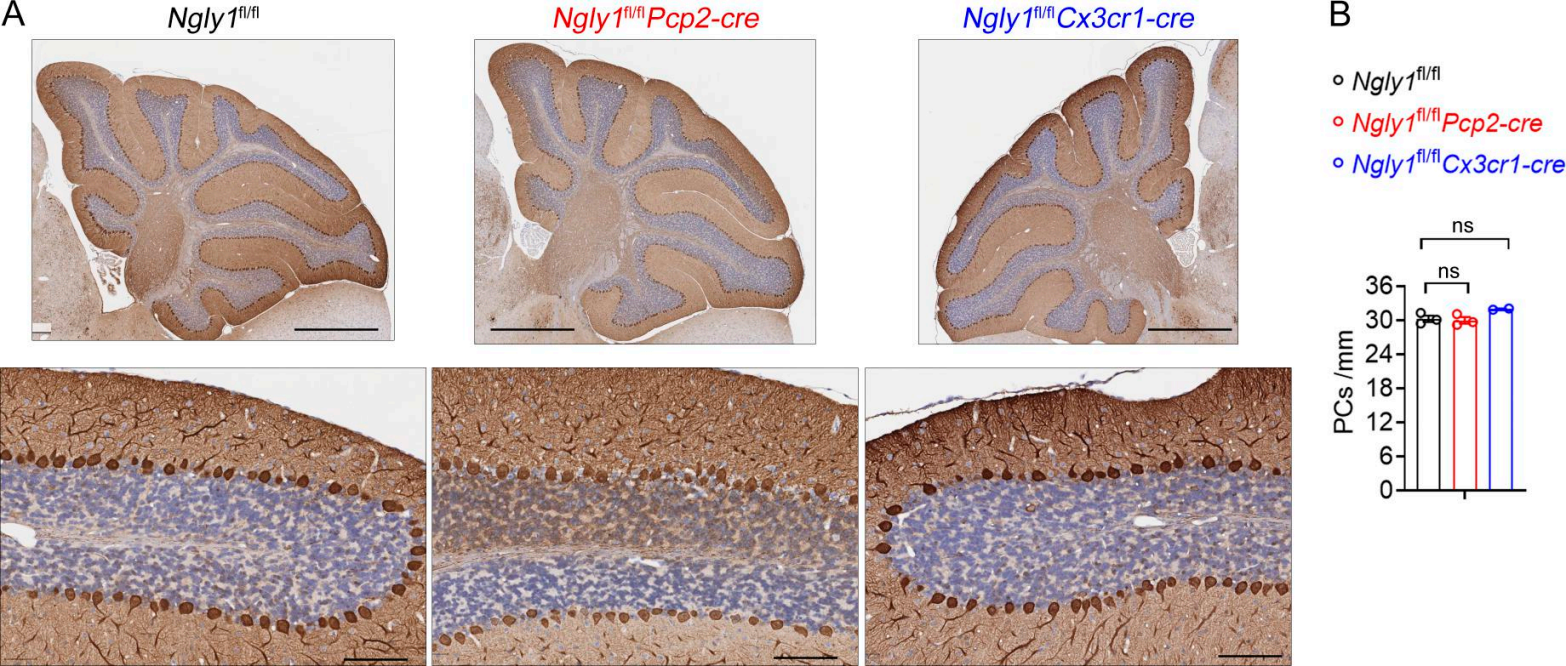
**Deletion of *Ngly1* in Purkinje cells or microglia alone did not cause neurodegeneration. (A)** Representative IHC staining of Purkinje cell marker calbindin in cerebella of 6-mo-old *Ngly1*^fl/fl^, *Ngly1*^fl/fl^*Pcp2-cre*, and *Ngly1*^fl/fl^*Cx3cr1-cre* control mice. Scale bar, 1 mm (upper panel), 100 μm (lower panel). **(B)** Quantification of calbindin-immunoreactive Purkinje cells shown in A. Data are shown as the mean ± SEM. Unpaired Student’s *t* test. ns, not significant.

We further generated Purkinje cell– and microglia-specific *Ngly1* knockout mice. However, neither mouse model exhibited any signs of neurological defects up to 1 year of age (data not shown). Histopathological analysis revealed Purkinje cell numbers in these mice were comparable to control mice (Fig. S2, A and B). We confirmed these conditional knockout mice by genotyping, but we were not able to confirm cell type–specific loss of NGLY1 protein in tissues due to the lack of an NGLY1 antibody that works for mouse tissue. These findings suggest that *Ngly1* loss in either Purkinje cells or microglia alone is not sufficient to cause neuropathology associated with NGLY1 deficiency such as Purkinje cell loss.

### The STING pathway mediates neurological disease of i*Ngly1*^−/−^ mice

**Figure 4.**
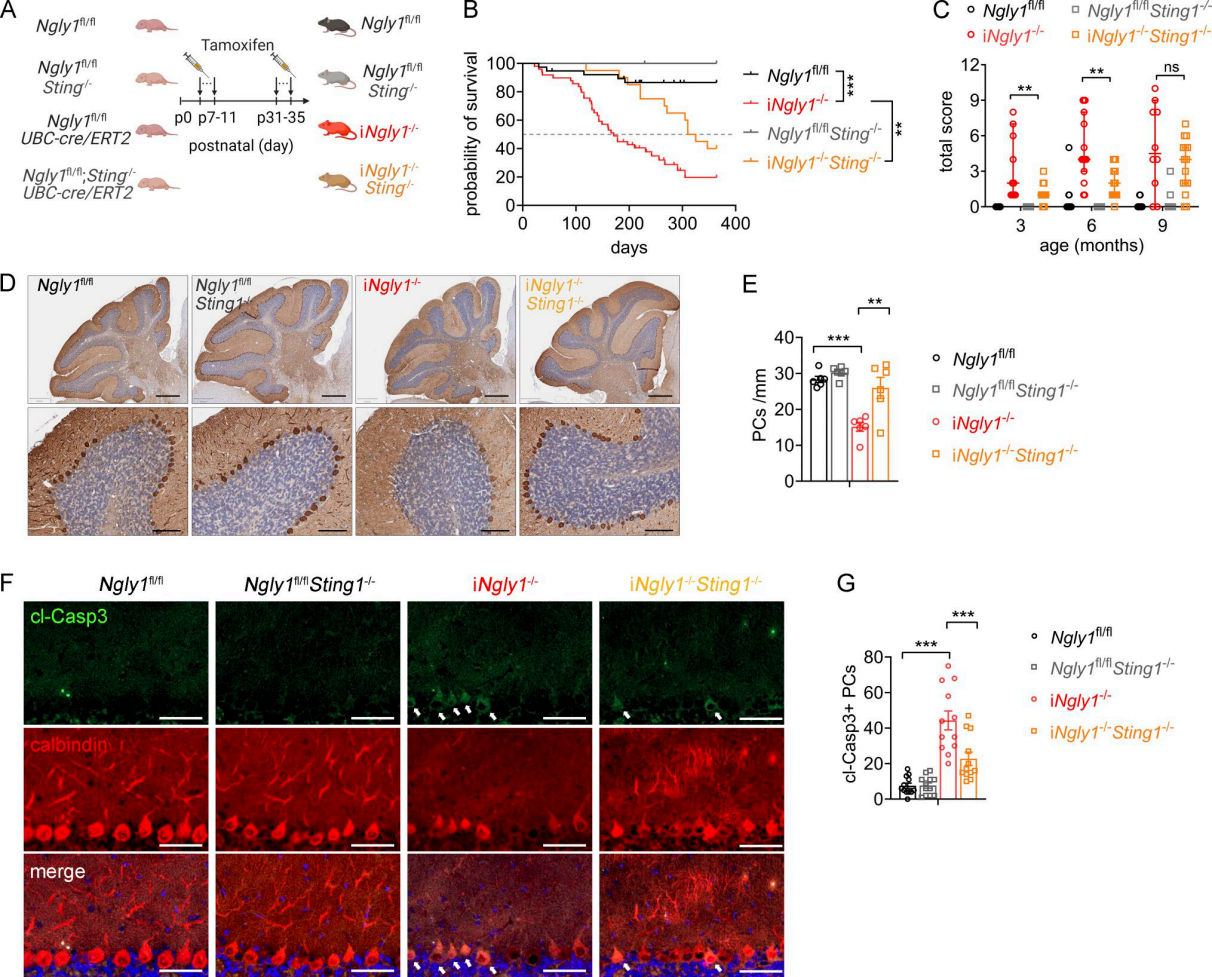
***Sting1*** ^**−/−**^ **ameliorates neurological disease of i*Ngly1*** ^**−/−**^ **mice. (A)** Schematic diagram showing generation of i*Ngly1*^−/−^*Sting1*^−/−^ mice. **(B)** Survival curves of *Ngly1*^fl/fl^, *Ngly1*^fl/fl^*Sting1*^−/−^, i*Ngly1*^−/−^, and i*Ngly1*^−/−^*Sting1*^−/−^ male mice. Log-rank (Mantel–Cox) test. **P < 0.01; ***P < 0.001. **(C)** Neurological deficit score of *Ngly1*^fl/fl^, *Ngly1*^fl/fl^*Sting1*^−/−^, i*Ngly1*^−/−^, and i*Ngly1*^−/−^*Sting1*^−/−^ male mice. Data were shown as median ± 95% CI. Mann–Whitney test. **(D)** IHC staining of calbindin in cerebella of 1-year-old *Ngly1*^fl/fl^, *Ngly1*^fl/fl^*Sting1*^−/−^, i*Ngly1*^−/−^, and i*Ngly1*^−/−^*Sting1*^−/−^ mice. Scale bar, 500 μm (upper), 100 μm (lower). **(E)** Quantification of Purkinje cell number of indicated genotypes. Data were shown as the mean ± SEM. One-way ANOVA with Bonferroni’s multiple comparisons test. **P < 0.01; ***P < 0.001. **(F and G)** IHC staining of cleaved caspase-3 (cl-Casp3, green) and calbindin (red) in cerebella of 8-wk-old *Ngly1*^fl/fl^, *Ngly1*^fl/fl^*Sting1*^−/−^, i*Ngly1*^−/−^, and i*Ngly1*^−/−^*Sting1*^−/−^ mice. Scale bar, 50 μm. Quantification of cl-Casp3^+^ Purkinje cells per midsagittal cerebellar section is shown in G. Data are from mice (*n* = 12 per genotype) of three independent experiments. Data were shown as the mean ± SEM. One-way ANOVA with Bonferroni’s multiple comparisons test. ***P < 0.001.

Our previous in vitro studies revealed that the absence of NGLY1 leads to cytosolic leakage of mitochondrial DNA (mtDNA), which activates cGAS-STING DNA sensing pathway in *Ngly1*^−/−^ cells. To investigate whether the STING pathway contributes to neuropathology of i*Ngly1*^−/−^ mice, we crossed *Ngly1*^fl/fl^*UBC-cre/ERT2* mice to *Sting1*^−/−^ and generated i*Ngly1*^−/−^*Sting1*^−/−^ mice via tamoxifen injection (Fig. 4 A). While about half of i*Ngly1*^−/−^ male mice died by postnatal day 170, the median survival of i*Ngly1*^−/−^*Sting1*^−/−^ male mice exceeded 300 days (Fig. 4 B). Additionally, i*Ngly1*^−/−^*Sting1*^−/−^ mice exhibited lower neurological disease score (improved motor function) compared with age- and sex-matched i*Ngly1*^−/−^ mice, particularly at younger ages (Fig. 4 C). Immunohistological analysis of the cerebellum showed that STING deletion prevented Purkinje cell loss in i*Ngly1*^−/−^ mice (Fig. 4, D and E). We also performed cleaved caspase-3 immunostaining on i*Ngly1*^−/−^*Sting1*^−/−^ mouse brain. We found that deletion of STING markedly reduces the number of cleaved caspase 3–positive Purkinje cells (Fig. 4, F and G). These genetic results suggest that the STING pathway plays a crucial role in the development of the neuropathology of NGLY1 deficiency.

### Single-nucleus transcriptomics of i*Ngly1*^−/−^ cerebellum

**Figure 5.**
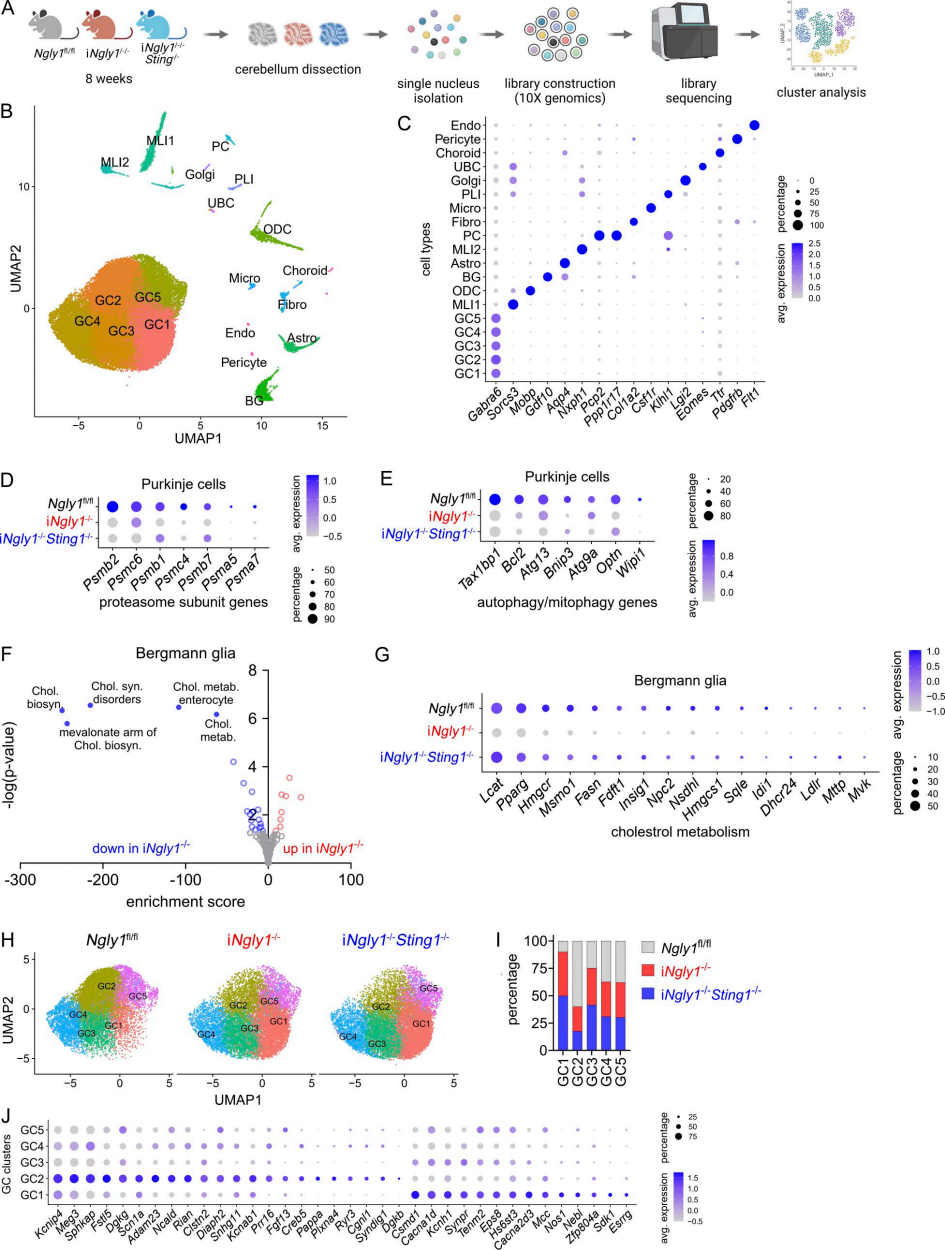
**snRNA-seq analysis of cells in i*Ngly1*** ^**−/−**^ **mouse cerebellum. (A)** Experimental workflow of snRNA-seq of 8-wk-old *Ngly1*^fl/fl^, i*Ngly1*^−/−^, and i*Ngly1*^−/−^*Sting1*^−/−^ mouse cerebella (*n* = 2 mice per genotype). **(B)** UMAP plot of all cells from i*Ngly1*^−/−^, i*Ngly1*^−/−^*Sting1*^−/−^, and *Ngly1*^fl/fl^ mouse cerebella. **(C)** Dot plots of cell type–specific marker genes. **(D and E)** Dot plots of proteasome subunit and autophagy/mitophagy-related genes in *Ngly1*^fl/fl^, i*Ngly1*^−/−^, and i*Ngly1*^−/−^*Sting1*^−/−^ Purkinje cells. **(F)** Pathway enrichment analysis of DEGs between *Ngly1*^fl/fl^ and i*Ngly1*^−/−^ Bergmann glia. **(G)** Dot plots of cholesterol metabolism–related genes in *Ngly1*^fl/fl^, i*Ngly1*^−/−^, and i*Ngly1*^−/−^*Sting1*^−/−^ Bergmann glia. **(H)** UMAP plot of granule cell subcluster from *Ngly1*^fl/fl^, i*Ngly1*^−/−^, or i*Ngly1*^−/−^*Sting1*^−/−^ mouse cerebella. **(I)** Quantification of three genotypes in each granule cell subcluster. **(J)** Dot plots of hallmark gene expression in GC1 and GC2 subclusters. UMAP, Uniform Manifold Approximation and Projection.

**Figure S3.**
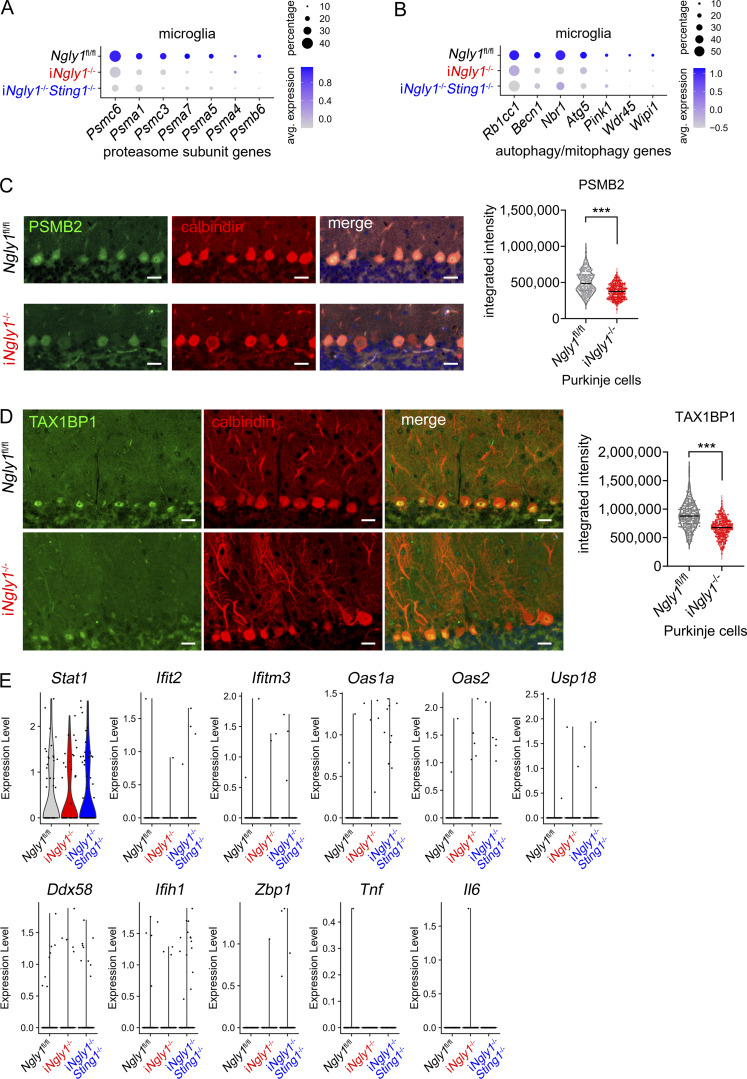
**snRNA-seq analysis of cells in i*Ngly1*** ^**−/−**^ **mouse cerebellum. (A and B)** Dot plots of proteasome subunit and autophagy/mitophagy-related gene expression in *Ngly1*^fl/fl^, i*Ngly1*^−/−^, and i*Ngly1*^−/−^*Sting1*^−/−^ mouse microglia. **(C and D)** Fluorescent IHC staining of PSMB2 and TAX1BP1 in 8-wk-old *Ngly1*^fl/fl^, i*Ngly1*^−/−^ cerebella. Scale bar, 20 μm. Fluorescence intensity per Purkinje cell of >200 cells from four mice per genotype was quantified and is shown on the right. Unpaired Student’s *t* test. ***P < 0.001. **(E)** Violin plots of ISG gene expression in *Ngly1*^fl/fl^, i*Ngly1*^−/−^, and i*Ngly1*^−/−^*Sting1*^−/−^ mouse microglia.

To explore the cell type–specific effects of *NGLY1* deficiency and the STING pathway in the cerebellum, we performed snRNA-seq of cerebella from *Ngly1*^fl/fl^, i*Ngly1*^−/−^, and i*Ngly1*^−/−^*Sting1*^−/−^ mice (Fig. 5 A). After stringent filtering, quality control, and unsupervised clustering analysis using the Seurat v3 R package, we identified 19 distinct clusters with unique transcriptional profiles (Fig. 5, B and C). We further analyzed differentially expressed genes (DEGs) between genotypes of each cell type. Consistent with bulk RNA data, deletion of *Ngly1* resulted in the reduced expression of proteasome subunit and autophagy/mitophagy-related genes in both Purkinje cells and microglia (Fig. 5, D and E; and Fig. S3, A and B), which is independent of STING, in line with previous findings showing these are NRF1 dependent (Yang et al., 2018). We further performed IHC staining for PSMB2 and TAX1BP1 and confirmed the downregulation of proteasome subunit and autophagy-related gene expression at the protein level in Purkinje cells (Fig. S3, C and D). We again did not observe any change in the expression of ISG or inflammatory cytokine genes in *iNgly1*^−/−^ microglia compared with *Ngly1*^fl/fl^ or i*Ngly1*^−/−^*Sting1*^−/−^ microglia, consistent with the lack of neuroinflammation in the *iNgly1*^−/−^ cerebellum (Fig. S3 E). These results validate our findings from the bulk RNA analysis.

**Figure S4.**
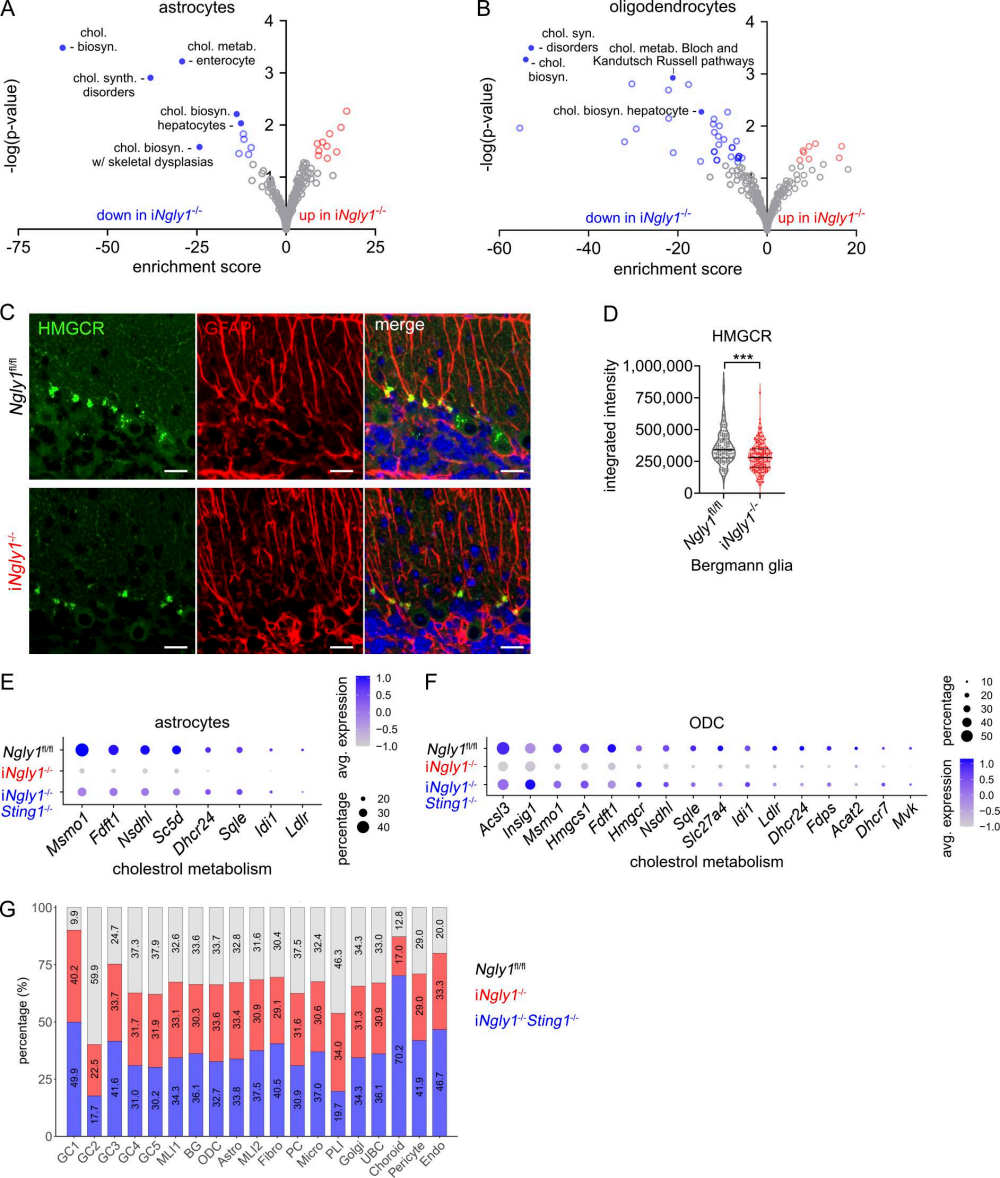
**Dysregulation of cholesterol biosynthesis in i*Ngly1*** ^**−/−**^ **mouse cerebellum. (A and B)** Pathway enrichment analysis of DEGs between *Ngly1*^fl/fl^ and i*Ngly1*^−/−^ astrocytes and oligodendrocytes. **(C and D)** Fluorescent IHC staining of HMGCR in 8-wk-old *Ngly1*^fl/fl^ and i*Ngly1*^−/−^ cerebella. Scale bar, 20 μm. Fluorescence intensity per Bergmann glia of >130 cells from four mice per genotype was quantified and shown in D. Unpaired Student’s *t* test. ***P < 0.001. **(E and F)** Dot plots of cholesterol metabolism–related genes in *Ngly1*^fl/fl^, i*Ngly1*^−/−^, and i*Ngly1*^−/−^*Sting1*^−/−^ astrocytes and oligodendrocytes (ODC). **(G)** Quantification of *Ngly1*^fl/fl^, i*Ngly1*^−/−^, and i*Ngly1*^−/−^*Sting1*^−/−^ (three genotypes in each cluster).

Further pathway enrichment analysis revealed that the cholesterol biosynthesis pathway was downregulated in i*Ngly1*^−/−^ across glial cell types, including Bergmann glia, astrocytes, and oligodendrocytes (Fig. 5 F and Fig. S4, A and B). IHC staining for HMGCR, a key enzyme in the cholesterol biosynthesis pathway, revealed reduced expression in Bergmann glia of i*Ngly1*^−/−^ mice compared with *Ngly1*^fl/fl^ controls (Fig. S4, C and D). This finding echoes the clinical observation that NGLY1 deficiency patients show reduced cholesterol levels (Lam et al., 2017). Interestingly, we found that i*Ngly1*^−/−^*Sting1*^−/−^ restored the expression of cholesterol biosynthesis genes in Bergmann glia back to wild-type levels (Fig. 5 G). In astrocytes and oligodendrocytes, we observed a similar reduction of cholesterol biosynthesis genes in i*Ngly1*^−/−^ mice that were partially rescued in i*Ngly1*^−/−^*Sting1*^−/−^ mice (Fig. S4, E and F). These data suggest that STING signaling represses cholesterol biosynthesis in i*Ngly1*^−/−^ cerebellar glial cells.

In addition to DEGs, we also observed cell population shift among the five granule cell subclusters between *Ngly1*^fl/fl^ and i*Ngly1*^−/−^ (Fig. 5, H and I; and Fig. S4 G). Compared with *Ngly1*^fl/fl^, i*Ngly1*^−/−^ exhibited an expansion of GC1 and a contraction of GC2 population and the shift was not reversed by *Sting1*^−/−^ (Fig. 5 I). While there is a lack of molecular classification of granule cells, we found GC1 and GC2 clusters have distinct expressions of ion channel genes; e.g., *Cacna1d*, *Cacna2d3*, *Kcnh1* are highly expressed in GC1, and *Kcinip4*, *Kcnab1*, *Scn1a* are highly expressed in GC2 (Fig. 5 J). Collectively, our snRNA-seq data reveal that Ngly1 loss leads to the decreased expression of proteasome and autophagy genes in Purkinje cells and a shift in granule cell subpopulations with different ion channel profiles, both of which are independent of STING. Ngly1 loss also causes decreased cholesterol biosynthesis in glial cells that is STING dependent.

**Figure S5.**
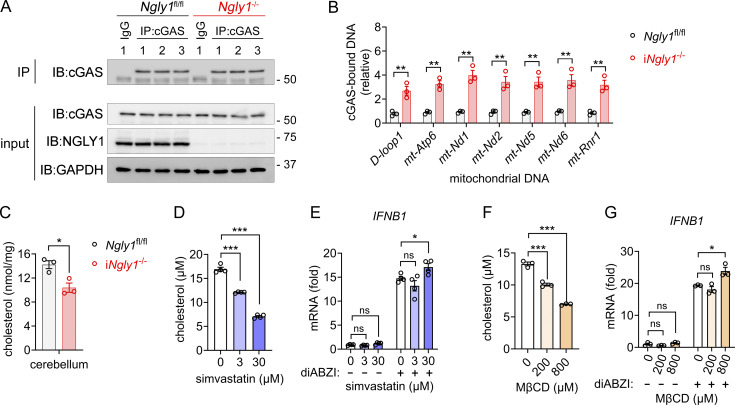
**Cytosolic release of mtDNA in i*Ngly1*** ^**−/−**^ **mouse cerebellum. (A)** Immunoprecipitation of endogenous cGAS in the *Ngly1*^fl/fl^, i*Ngly1*^−/−^ cerebellar lysate. *n* = 3 mice per genotype. **(B)** qPCR analysis of cGAS-bound mtDNA in the *Ngly1*^fl/fl^, i*Ngly1*^−/−^ cerebellar lysate. *n* = 3 mice per genotype. Data are the mean ± SEM. Unpaired Student’s *t* test. **P < 0.01. **(C)** Quantification of cholesterol in *Ngly1*^fl/fl^, i*Ngly1*^−/−^ cerebella. *n* = 3 mice per genotype. Data are the mean ± SEM. Unpaired Student’s *t* test. *P < 0.05. **(D)** Quantification of cholesterol in SK-N-SH cells treated with simvastatin at indicated concentrations for 24 h. Data are the mean ± SEM of three biological replicates. Unpaired Student’s *t* test. ***P < 0.001. **(E)** qRT-PCR analysis of *IFNB1* gene expression in simvastatin-treated SK-N-SH cells after with or without diABZI (2 µM) treatment for 2 h. Data are the mean ± SEM of three biological replicates. Unpaired Student’s *t* test. *P < 0.05; ns, not significant. **(F)** Quantification of cholesterol in SK-N-SH cells treated with cholesterol-depleting agent MβCD at indicated concentrations for 48 h. Data are the mean ± SEM of three biological replicates. Unpaired Student’s *t* test. ***P < 0.001. **(G)** qRT-PCR analysis of *IFNB1* gene expression in MβCD-treated SK-N-SH cells after with or without diABZI (2 µM) treatment for 2 h. Data are the mean ± SEM of three biological replicates. Unpaired Student’s *t* test. *P < 0.05; ns, not significant. Source data are available for this figure: SourceData FS5.

We next investigated potential mechanisms underlying STING activation in i*Ngly1*^−/−^ mice. To test whether mtDNA leakage contributes to activation of the cGAS-STING pathway, we conducted cGAS immunoprecipitation followed by quantitative PCR (qPCR) to quantify cGAS-bound mtDNA in cerebellar tissue (Fig. S5 A). We observed increased levels of cGAS-bound mtDNA in the *Ngly1*-deficient cerebellum, supporting a role of mtDNA leakage in STING activation (Fig. S5 B). One previous study suggested a link between cholesterol biosynthesis inhibition and type I IFN responses via STING (York et al., 2015). We indeed observed reduced cholesterol level in i*Ngly1*^−/−^ cerebellar tissue compared with *Ngly1*^fl/fl^ (Fig. S5 C). We further explored whether reduced cholesterol might contribute to STING activation. We used neuroblastoma cell line SK-N-SH, which has an intact STING pathway. We reduced cellular cholesterol levels using either the HMG-CoA reductase inhibitor simvastatin or the cholesterol-depleting agent methyl-β-cyclodextrin (MβCD) and then examined IFN pathway activation with or without STING agonist (diABZI) treatment (Fig. S5, D and F). Reduced cholesterol did not induce a spontaneous IFN response nor enhance STING activation (Fig. S5, E and G). Together, these results suggest that mitochondrial mtDNA leakage is a likely mechanism for STING activation in Purkinje cells in i*Ngly1*^−/−^ mice.

### An orally bioactive STING antagonist and treatment of i*Ngly1*^−/−^ mice

**Figure 6.**
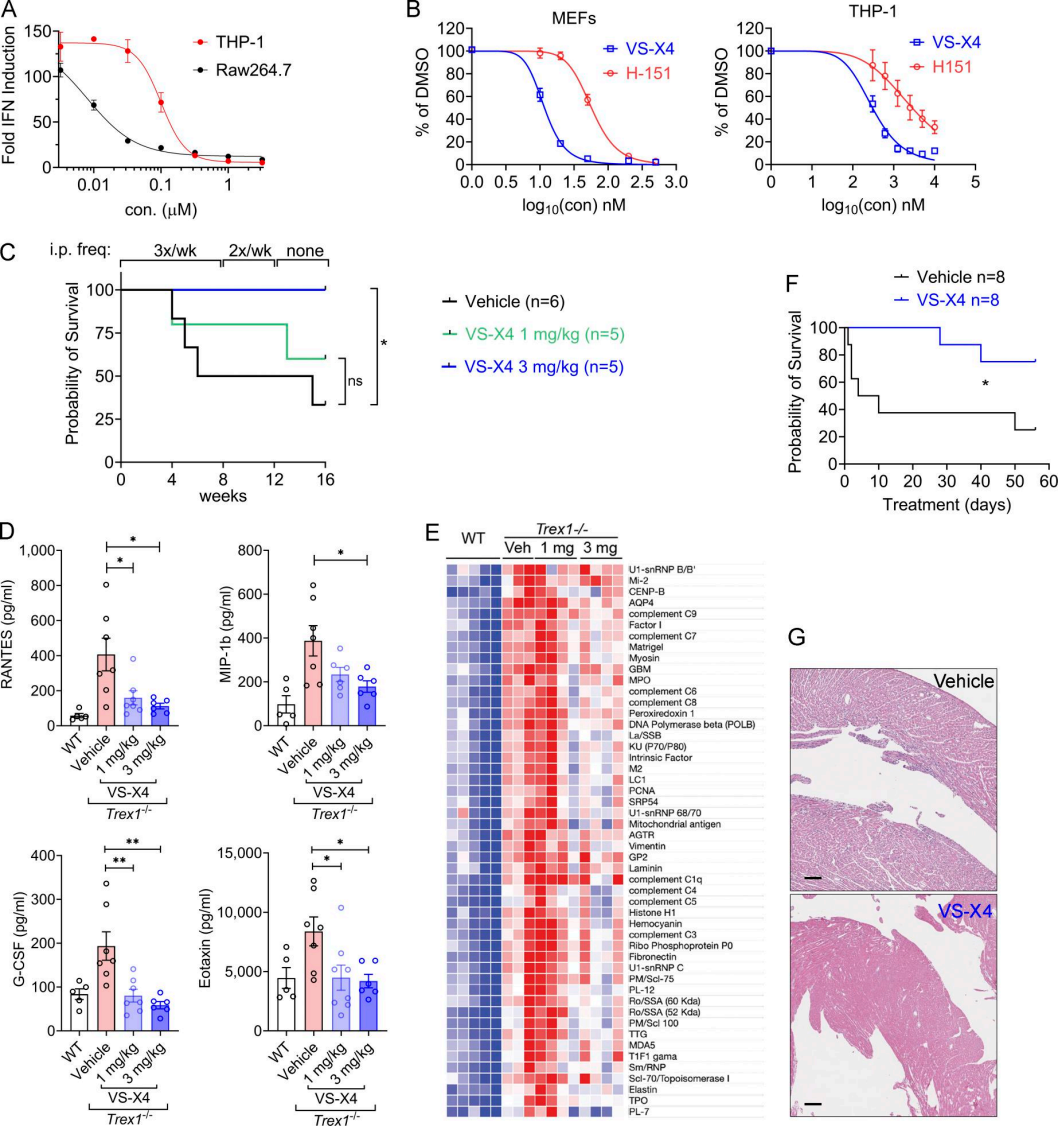
**Orally bioactive STING antagonist VS-X4 ameliorates STING-mediated autoimmune disease. (A)** IC_50_ of VS-X4 in THP-1 and RAW264.7 cells treated with 10 μM 2′-5′-cGAMP. **(B)** IC_50_ of VS-X4 and H-151 in MEFs or THP-1 treated with 10 μM 2′-5′-cGAMP. **(C)** Survival of *Trex1*^−/−^ mice treated with vehicle or VS-X4 intraperitoneally at the indicated doses. Log-rank (Mantel–Cox) test. *P < 0.05; ns, not significant. **(D)** Serum cytokines in *Trex1*^−/−^ mice treated with vehicle or VS-X4 were measured at 8 wk after treatment. Data are shown as the mean ± SEM. **(E)** Unpaired Student’s *t* test. *P < 0.05; **P < 0.01. (E) Serum autoantibodies in *Trex1*^−/−^ mice treated with vehicle or VS-X4 were analyzed using an autoantibody microarray. **(F)** Survival of *Trex1*^−/−^ mice treated with vehicle or VS-X4 orally. Log-rank (Mantel–Cox) test. *P < 0.05. **(G)** H&E staining of *Trex1*^−/−^ mouse hearts treated with vehicle or VS-X4.

There is an emerging interest in developing STING antagonists for treating inflammatory and neurological diseases (Barasa et al., 2023; Haag et al., 2018; Hong et al., 2021). We tested a recently developed STING antagonist VS-X4 (Materials and methods). VS-X4 potently inhibited cGAMP-induced IFN response in THP-1 human monocytes with an IC_50_ of 100.1 nM and in RAW264.7 mouse macrophages with an IC_50_ of ∼8 nM (Fig. 6 A). VS-X4 was also more effective when compared to a commonly used STING inhibitor H-151 in both mouse and human cells (Fig. 6 B).

To assess the in vivo activity, we first tested VS-X4 via intraperitoneal injection in *Trex1*^−/−^ mice, a model of STING-mediated systemic autoimmune disease (Ahn et al., 2014). VS-X4 treatment significantly improved overall survival and markedly reduced both autoantibodies and inflammatory cytokines in the serum (Fig. 6, C–E). We further tested its oral bioavailability and found that the oral administration of VS-X4 also significantly improved overall survival and reduced tissue inflammation in the hearts of *Trex1*^−/−^ mice (Fig. 6, F and G).

**Figure 7.**
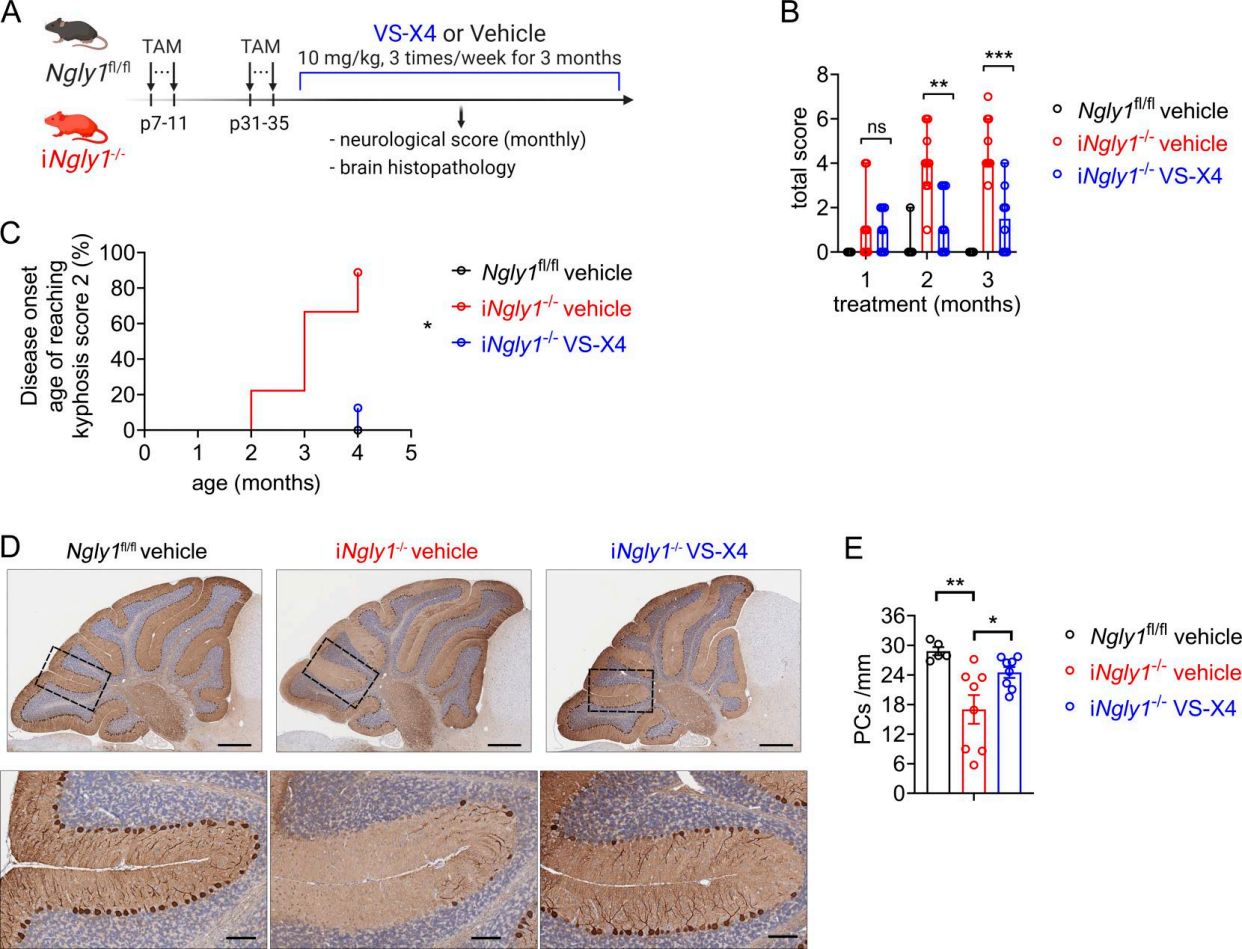
**Pharmacological inhibition of STING ameliorates neurological disease of NGLY1 deficiency. (A)** Experimental workflow of treatment of i*Ngly1*^−/−^ mice. **(B)** Neurological deficit score of *Ngly1*^fl/fl^ (*n* = 7 mice) and i*Ngly1*^−/−^ mice treated with STING inhibitor VS-X4 (*n* = 8 mice) or vehicle control (*n* = 9 mice). Data were shown as median ± 95% CI. Mann–Whitney *U* test. **P < 0.01; ***P < 0.001; ns, not significant. **(C)** Disease onset (disease incidence in the month when kyphosis score reaches 2) of *Ngly1*^fl/fl^ (*n* = 7 mice) and i*Ngly1*^−/−^ mice treated with STING inhibitor VS-X4 (*n* = 8 mice) or vehicle control (*n* = 9 mice). Log-rank (Mantel–Cox) test. *P < 0.05. **(D)** IHC staining of calbindin in cerebella of i*Ngly1*^−/−^ mice treated with the STING inhibitor VS-X4 or vehicle control for 3 mo. Scale bar, 500 μm (upper), 100 μm (lower). **(E)** Quantification of Purkinje cell number of i*Ngly1*^−/−^ mice treated with the STING inhibitor VS-X4 or vehicle control for 3 mo. One-way ANOVA with Bonferroni’s multiple comparisons test. *P < 0.05; **P < 0.01.

We next treated i*Ngly1*^−/−^ mice orally with VS-X4 for 3 mo, starting at 1-mo-old (Fig. 7 A). The motor function of i*Ngly1*^−/−^ mice improved significantly after just 2 mo of VS-X4 treatment (Fig. 7 B). The disease onset, as determined by kyphosis, was delayed in VS-X4–treated mice (Fig. 7 C). IHC analysis of cerebellum revealed significantly increased number of Purkinje cells in VS-X4–treated mice compared with vehicle-treated controls (Fig. 7, D and E). Together, these results provide preclinical evidence that targeting the STING pathway with an orally bioactive antagonist could be an effective treatment for NGLY1 deficiency and potentially for many other STING-associated neurodegenerative diseases.

## Discussion

We present a *Ngly1*-deficient mouse model on the C57BL/6 background that is genetically trackable and develops progressive neurodegenerative disease. Previously reported mouse models of Ngly1 are either embryonic lethal (germline knockout on the C57BL/6 background) (Fujihira et al., 2017) or present nonprogressive disease and incompatible with genetic studies (germline knockout on C57BL/6-JF1 mixed background) (Asahina et al., 2021). These earlier models played an important role in understanding NGLY1 biology, although they are limited in genetic and pharmacological studies critical for therapeutic development for this rare disease. Using the *iNgly1* mouse model, we provide genetic and pharmacological evidence to demonstrate that the STING pathway plays a crucial role in the development of neurological disease associated with *Ngly1* deficiency in mice.

Our study provides several new insights into the mechanism of NGLY1 disease. The most notable phenotype is Purkinje cell loss, which we observed in both viable *Ngly1* knockout mouse models and in postmortem brain tissue of a human NGLY1 patient. Though NGLY1 is thought to be a housekeeping enzyme expressing ubiquitously across cell types, we find that Purkinje cells exhibit the highest expression of NGLY1 compared with other neural cell types in the brain. We previously showed that STING is also expressed in Purkinje cells in the mouse cerebellum (Chu et al., 2021). The gradual loss of Purkinje cells after postnatal whole-body *Ngly1* gene deletion is also consistent with the progressive worsening of motor and coordination of these mice. However, tissue-specific deletion of *Ngly1* in Purkinje cells or microglia alone is insufficient to induce Purkinje cell loss or neurological disease. Therefore, the selective expression of NGLY1 and STING in Purkinje cells may render these neurons more susceptible to NGLY1 loss, but signals from other cell types are also required for the development of neuropathology.

snRNA-seq analysis provided important clues on what other signals may be present in the cerebellum of *iNgly1*^−/−^ mice. (1) We observed a shift in two granule cell subpopulations, from GC2 (high potassium channel expression) to GC1 (high calcium channel expression). Granule cells are the most abundant neurons in the cerebellum, which provide primary excitatory input to Purkinje cells. It is unclear what are the functional differences between these two subpopulations of granule cells, although one possibility is that this shift in ion channel profiles could disrupt signal inputs that support Purkinje cell function. (2) We found that the cholesterol biosynthesis pathway is downregulated in *iNgly1*^−/−^ Bergmann glia, astrocytes, and oligodendrocytes compared with both wild-type and *iNgly1*^−/−^*Sting1*^−/−^. This is important because reduced cholesterol level is a clinical symptom of NGLY1 patients observed in multiple clinical studies (Lam et al., 2017). Multi-omics studies of NGLY1 patient–derived plasma metabolites also identified reduced cholesterol and other lipids (Chen et al., 2021, *Preprint*). Also, STING signaling activation has been previously shown to repress cholesterol biosynthesis, through either IFN signaling or sterol O-acyltransferase 1–dependent cholesterol esterification (York et al., 2015; Zhang et al., 2024). In addition, ER-bound transcription factor NRF1, which requires NGLY1 deglycosylation for its activity, has been proposed as a sensor of cholesterol homeostasis (Widenmaier et al., 2017). Thus, it is possible that Ngly1 loss leads to reduced cholesterol biosynthesis due to STING-mediated repression and/or inactive NRF1, which may metabolically impact glial cell function. (3) In Purkinje cell population, we found reduced expression of proteasome genes and autophagy/mitophagy genes. These changes have been observed in vitro due to reduced NRF1 transcriptional activity associated with NGLY1 loss (Galeone et al., 2020; Lehrbach et al., 2019; Tomlin et al., 2017; Ward et al., 2024; Yang et al., 2018). The reduced capacity in protein degradation and organelle turnover could also negatively impact Purkinje cell function. Taken together, the development of neurological disease in *iNgly1*^−/−^ mice is likely due to both Purkinje cell–intrinsic and cell-extrinsic signals. Uncovering precisely how these signals integrate and then lead to progressive loss of motor function in *iNgly1*^−/−^ mice remains an important area for future investigation.

We did not observe signs of systemic inflammation or neuroinflammation in *iNgly1*^−/−^ cerebellum by transcriptome or immunohistochemistry analysis. This seems to be at odds with our genetic evidence supporting STING playing a crucial role in disease development. However, we and others have shown that STING has many IFN and IFN-independent signaling activities that are cell type specific (Wu et al., 2020). For example, STING activity in myeloid cells is IFN-dependent, while STING activity in lymphoid cells is IFN-independent. IFN-independent activities of STING include autophagy (Gui et al., 2019), inflammasome activation (Gaidt et al., 2017), and function as a proton channel (Liu et al., 2023; Xun et al., 2024). Based on our genetic and snRNA-seq data, it is possible that STING represses cholesterol biosynthesis in cerebellar glial cells, which contributes to the development of NGLY1 disease. Of note, studies of mixed background germline *Ngly1* knockout mice and *Ngly1* knockout rats did reveal neuroinflammation in the thalamus and hippocampus region (Asahina et al., 2021; Asahina et al., 2020). It is possible that the postnatal timing of *Ngly1* deletion is the reason for the discrepancy in region-specific neuroinflammation between different Ngly1 models. From the standpoint of STING biology, the *iNgly1*^−/−^ mouse stands as a unique model to evaluate the role of the STING pathway in neurodegeneration without confounding neuroinflammation.

In summary, our work provides direct mouse genetic evidence supporting the contribution of the STING pathway to the neurodegeneration associated with NGLY1 deficiency. The proof of concept demonstrated by targeting the STING pathway with an orally bioactive STING antagonist VS-X4 paves new avenues for therapeutic interventions, not only for NGLY1 deficiency but also for other neurodegenerative diseases involving the STING pathway.

## Materials and methods

### Animals

*Ngly1* ^fl/fl^ C57BL/6J mice were kind gifts from Dr. Tadashi Suzuki (RIKEN, Saitama, Japan) (Fujihira et al., 2020) and crossed with hemizygous B6.Cg-*Ndor1*^*Tg(UBC-cre/ERT2)1Ejb*^/1J transgenic mice (*UBC-Cre-ERT2*, Strain #007001; Jackson Laboratories), which express a Cre-ERT2 fusion gene under the control of the human ubiquitin C (UBC) promoter. *Sting1*^−/−^ (C57BL/6J) were provided by Dr. Glen Barber (University of Miami, Miami, FL, USA). *Trex1*^−/−^ mice were maintained as described previously (Hasan et al., 2015). B6.129-Tg(Pcp2-cre)2Mpin/J (*Pcp2-cre*, Strain #: 004146; JAX) and B6J.B6N(Cg)-*Cx3cr1*^*tm1.1(cre)Jung*^/J (*Cx3cr1-cre*, Strain #: 025524; JAX) mice were purchased from Jackson Laboratories.

To generate postnatal tamoxifen-inducible *Ngly1* knockout mice (i*Ngly1*^−/−^), *Ngly1*^fl/fl^*UBC-Cre-ERT2* pups and their *Ngly1*^fl/fl^ littermates were injected with 100 μg tamoxifen (cat #T5648; Sigma-Aldrich) in corn oil intraperitoneally at postnatal day 7 (P7) for 5 consecutive days. After weaning, mice were injected with tamoxifen (75 mg/kg body weight) intraperitoneally for 5 consecutive days. *Ngly1*^fl/fl^*UBC-Cre-ERT2* and derivative i*Ngly1*^−/−^ mice were housed in specific pathogen-free barrier facilities at the University of Texas Southwestern (UT Southwestern) Medical Center. The animal protocol was approved by the Institutional Animal Care and Use Committee at UT Southwestern Medical Center (APN 2017-101968). *Ngly1*^−/−^ (JF1/B6F1) mice and their isogenic *Ngly1*^+/+^ controls were generated as described previously (Asahina et al., 2021), and the animal procedures were approved by either the Institutional Committee of RIKEN (approval no. H28-2-003(2)) or the Experimental Animal Care and Use Committee of Takeda Pharmaceutical Co., Ltd.

Wild-type nonhuman primate cynomolgus macaque monkeys (22–26 mo old) were purchased and maintained at a study-level quarantine facility. The monkeys were singly housed in appropriately sized caging in accordance with the USDA Animal Welfare Act (9 CFR, Parts 1, 2, and 3). Following euthanasia, the animals will be perfused with chilled 0.9% saline for at least 5 min or until the fluid runs sufficiently clear. Slabbed brain samples will be collected and preserved in 10% neutral buffered formalin for immunohistochemistry.

### NGLY1 deficiency patient specimen

Formalin-fixed paraffin-embedded cerebellum of one deidentified NGLY1 deficiency patient (Stuut et al., 2021) was obtained from Stanford Biobank. Age-matched cerebellum slides from one deidentified healthy control were obtained from Department of Pathology at UT Southwestern Medical Center. No Institutional Review Board approval was needed for deidentified samples.

### Reagents

Tamoxifen (cat #T5648) and corn oil (cat #C8267) were from Sigma-Aldrich. 2′3′-cGAMP (cat #tlrl-nacga23-5) was from InvivoGen. THP-1-Lucia ISG cells and RAW-Lucia ISG cells were from InvivoGen. VS-X4, a small molecule heterocycle, was designed and synthesized at Spring Bank Pharmaceuticals, Inc. Simvastatin (cat #HY-17502) and MβCD (cat #HY-101461) were from MedChemExpress. The Cholesterol/Cholesterol Ester-Glo Assay kit was from Promega (cat #J3190).

### Cell culture

SK-N-SH cells were purchased from the ATCC (HTB-11; ATCC) and maintained in DMEM with 10% (vol/vol) FBS, 10 mM HEPES, 2 mM L-glutamine, and 1 mM sodium pyruvate with the addition of 100 U/ml penicillin and 100 mg/ml streptomycin, at 37°C with 5% CO_2_. Cells were tested negative for *Mycoplasma*. In simvastatin or MβCD treatment, SK-N-SH cells were cultured in serum-free medium and treated with simvastatin or MβCD for indicated time, then stimulated with diABZI (2 mM) for 2 h. The cell lysate and RNA samples were collected for cellular cholesterol and qRT-PCR analysis, respectively.

### Mouse motor function tests

Motor function of i*Ngly1*^−/−^ mice was evaluated using a composite phenotype scoring system as described previously (Guyenet et al., 2010). Briefly, mice were assessed using four measures including the hind ledge test, limb clasping, gait, and kyphosis. Each measure is scored on a scale of 0–3, with a total score of 0–12 for all four measures.

### Histopathology and immunohistochemistry

H&E staining of mouse tissues was performed in UT Southwestern Medical Center HistoPathology Core. Fluorescent immunohistochemistry was performed in UT Southwestern Medical Center Tissue Management Shared Resource Core. H&E staining of NGLY1 deficiency patient’s cerebellum was performed as described previously (Stuut et al., 2021). Primary antibodies used for immunohistochemistry are as follows: Monoclonal Anti-Calbindin-D-28K antibody (cat #C9848, clone CB-955,1:1,000 dilution; Sigma-Aldrich); Anti-Iba1/AIF1 Antibody (cat #MABN92, 1:400 dilution; MilliporeSigma); Cleaved Caspase-3 (Asp175) Antibody (cat #9661, 1:200 dilution; Cell Signaling); Anti-COX IV antibody (cat #ab16056, 1 µg/ml; Abcam); PSMB2 Antibody (cat #ab236752, 1:200 dilution; Abcam), TAX1BP1 Polyclonal antibody (cat #14424-1-AP, 1:250 dilution; Proteintech), HMGCR antibody (cat #LS-B16059, 1:100 dilution; LifeSpan BioSciences). Fluorophore-conjugated secondary antibodies were used as follows: Goat anti-Rabbit IgG (H+L) Highly Cross-Adsorbed Secondary Antibody, Alexa Fluor Plus 488 (cat #A32731, 1:200 dilution; Thermo Fisher Scientific); Donkey anti-Mouse IgG (H+L) Highly Cross-Adsorbed Secondary Antibody, Alexa Fluor 546 (cat #A10036, 1:200 dilution; Thermo Fisher Scientific); Donkey anti-Mouse IgG (H+L) Highly Cross-Adsorbed Secondary Antibody, Alexa Fluor 647 (cat #A-31571, 1:500 dilution; Thermo Fisher Scientific). Nuclei were counterstained with DAPI. Immunofluorescence images were acquired using a Zeiss LSM 780 or 880 confocal microscope (Zeiss) in UT Southwestern Medical Center Live Cell Imaging Core or using Zeiss AxioScan.Z1 in UTSW Whole Brain Microscopy Facility. DAB immunohistochemistry of calbindin (Calbindin [D1I4Q] XP Rabbit mAb, cat #13176; Cell Signaling) was performed at HistoWiz. Calbindin-immunoreactive Purkinje cells were quantified using QuPath-0.3.2 software. For NHP brain FFPE slices, IHC was performed using a Leica Bond automated immunostainer and a rabbit anti-NGLY1 antibody (cat #HPA036825, 1:100 dilutions; Atlas Antibodies). DAB immunohistochemistry was used to visualize NGLY1 immunoreactivity. ImageJ and QuPath software were used to quantify microscopic data.

### Western blotting

Western blotting was performed as previously described (Yang et al., 2018). Briefly, homogenized tissues from i*Ngly1*^−/−^ and *Ngly1*^fl/fl^ mice were lysed in radioimmunoprecipitation assay buffer, and tissue lysates were quantified using BCA. Equal amounts of proteins were separated on SDS-PAGE and transferred to the nitrocellulose membrane. Blotting membranes were blocked with 5% nonfat milk and incubated with diluted primary antibodies at 4°C overnight according to the manufacturers’ recommendation. Membranes were further incubated with HRP-conjugated secondary antibody (Bio-Rad), and SuperSignal West Pico Chemiluminescent Substrate (Thermo Fisher Scientific) was used to develop the blots either on an x-ray film or using ChemiDoc imaging system (Bio-Rad). Primary antibodies used were as follows: NGLY1 mouse monoclonal antibody as described previously (Yang et al., 2018), anti-HMGB1 antibody (cat #ab18256, 1:2,000 dilution; Abcam), anti-GAPDH (cat #2118, 1:2,000 dilution; Cell Signaling).

### Immunoprecipitation of endogenous cGAS and DNA analysis

Immunoprecipitation of endogenous cGAS was performed as previously described (Tu et al., 2022). Briefly, freshly dissected mouse cerebellum was homogenized in immunoprecipitation lysis buffer (20 mM Tris-HCl, pH 7.4, 0.5% Nonidet P-40, 150 mM NaCl, and 1× protease inhibitor mixture) and centrifuged at 20,000 × *g* for 20 min at 4°C to remove debris. cGAS antibody (ab252416, 0.3 µg; Abcam) was incubated with 1 mg of tissue lysate with rotation at 4°C overnight. Protein G Dynabeads (10004D; 50 μl; Thermo Fisher Scientific) was incubated with the lysate and antibody mix for 1 h with rotation at 4°C. After wash, cGAS-bound DNA was isolated using QIAamp DNA Mini Kit (56304; Qiagen) and analyzed using qPCR. Primer sequences were reported previously (Tu et al., 2022).

### RNA isolation and quantitative RT-PCR

Total RNA was isolated from homogenized mouse tissue or cultured cells using TRI reagent (Sigma-Aldrich), and cDNA was synthesized using iScript cDNA Synthesis kit (Bio-Rad). qPCR was performed using iTaq Universal SYBR Green Supermix (Bio-Rad) per the manufacturer’s instruction. Primer sequences used were described previously (Yang et al., 2018). Heatmap of gene expression was generated using Multiple Experiment Viewer (MeV4).

### Mouse treatment

i*Ngly1*^−/−^ and *Ngly1*^fl/fl^ were separated and assigned to vehicle or VS-X4 treatment groups after weaning. For STING inhibitor VS-X4 oral dosing, VS-X4 powder was mixed with peanut butter to reach the indicated drug concentration (10 mg/kg body weight). Mice were fed with 200 μl of either plain peanut butter (vehicle control) or peanut butter containing VS-X4 three times per week for 3 mo. During the treatment, mice were evaluated every month using a composite phenotype scoring system (Guyenet et al., 2010). At the end of 3-mo treatment, mice were sacrificed and brains were perfused and fixed for IHC staining.

For VS-X4 intraperitoneal treatment of *Trex1*^−/−^ mice, VS-X4 was dissolved in DMSO and formulated at DMSO:PEG400:H_2_O 5:20:75 (volume). VS-X4 was injected intraperitoneally at the indicated dose. Serum was collected after 8-wk VS-X4 treatment. Serum autoantibody and inflammatory cytokines were analyzed as described previously (Hasan et al., 2015; Tu et al., 2022). For oral gavage, VS-X4 was formulated at DMSO:PEG400:H_2_O 5:20:75 (volume). After 8-wk VS-X4 oral treatment, mouse hearts were fixed for histopathology.

### snRNA-seq and data analysis

Eight-wk-old *Ngly1*^fl/fl^, i*Ngly1*^−/−^, and i*Ngly1*^−/−^*Sting1*^−/−^ male mice (two mice/genotype) were sacrificed, and cerebella were collected and frozen in liquid nitrogen. Nuclei were isolated from frozen cerebella as described previously (Ayhan et al., 2021). Briefly, cerebellum tissues were homogenized in 2 ml of ice-cold Nuclei EZ lysis buffer (cat #EZ PREP NUC-101; MilliporeSigma) using Dounce homogenizers. Nuclei were centrifuged at 500 × *g* at 4°C for 5 min and washed with ice-cold Nuclei EZ lysis buffer. After wash, the nuclei were resuspended in nuclei suspension buffer (NSB) consisting of 1× PBS, 1% BSA (cat #AM2618; Thermo Fisher Scientific), and 0.2 U/μl RNase inhibitor (cat #AM2694; Thermo Fisher Scientific) and were filtered through 40-μm Flowmi Cell Strainer (cat #H13680-0040; Bel-Art). Cell suspension was adjusted to a final concentration of 1,000 nuclei/μl with NSB. Samples of each cerebellum were prepared separately and barcoded during library preparation. Droplet-based snRNA sequencing was performed in the McDermott Center Next Generation Sequencing Core at UT Southwestern Medical Center. snRNA-seq libraries were prepared using the Chromium Single Cell 5′ Reagent Kits (10x Genomics) according to the manufacturer’s protocol. Libraries were sequenced using an Illumina NovaSeq 6000. Reads were aligned to the mouse reference mm10-2020-A using CellRanger 7 software (10x Genomics).

Clustering analysis was performed in the Neuroinformatics Core at UT Southwestern Medical Center as described previously (Ayhan et al., 2021). R package Seurat (v3.2.0) and custom scripts were used to identify individual clusters. Nuclei with UMI > 800, 500 < nCount < 200,000, and mitochondrial transcripts <0.1% were retained for downstream analysis. Each dataset was log-normalized with a scale factor of 10,000 using NormalizeData, and the top 2,000 variable genes were identified with FindVariableFeatures. A resolution of 0.4 was used to identify major clusters and for Uniform Manifold Approximation and Projection. Clusters were merged based on shared expression of canonical cell-type markers, and one cluster with a disproportionately high percentage of mitochondrial genes was excluded for further analysis. Differential expression analysis between genotypes of each cluster was performed with the Wilcoxon rank sum test using Seurat’s FindAllMarkers function. Pathway enrichment analysis of significantly DEGs was performed with Enrichr (Xie et al., 2021).

### Statistical analysis

GraphPad Prism was used for statistical analysis. Statistical tests performed were indicated in figure legends. P values <0.05 were considered statistically significant.

### Online supplemental material

Fig. S1 shows additional characterization of Ngly1-deficient mice. Fig. S2 shows mouse models of cell type–specific deletion of *Ngly1* in Purkinje cells or microglia. Figs. S3 and S4 show additional snRNA-seq analysis of cells in i*Ngly1*^−/−^ mouse cerebellum and validation. Fig. S5 shows mtDNA leakage in i*Ngly1*^−/−^ mouse cerebellum and the effect of cholesterol dysregulation on the STING pathway. Video 1 shows motor defect of i*Ngly1*^−/−^ mice.

## Supplementary Material

SourceData F1 is the source file for Fig. 1.

SourceData FS5 is the source file for Fig. S5.

## Data Availability

The snRNA-seq data (related to Figs. 5, S3, and S4) have been deposited on GEO under the accession number GSE295078. Fully uncropped and unprocessed images for each blot are provided in the source data.
