# Supplementary figures and images for: The STING pathway drives noninflammatory neurodegeneration in NGLY1 deficiency
Source: J Exp Med. 2025 Jul 11;222(10):e20242296. doi: 10.1084/jem.20242296 (PMC12249164; doi:10.1084/jem.20242296)

Source data: Figure 1

Figure 1B:

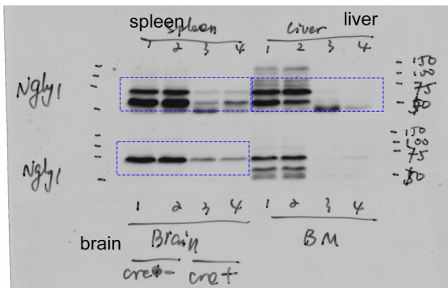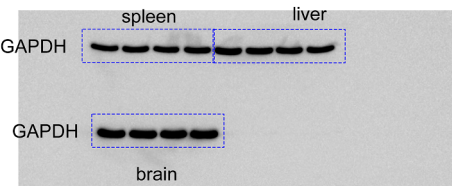

Supplement: SourceData F1 — is the source file for Fig. 1. [file jem_20242296_sourcedataf1.pdf]

Source data: Figure S5

Figure S5A:

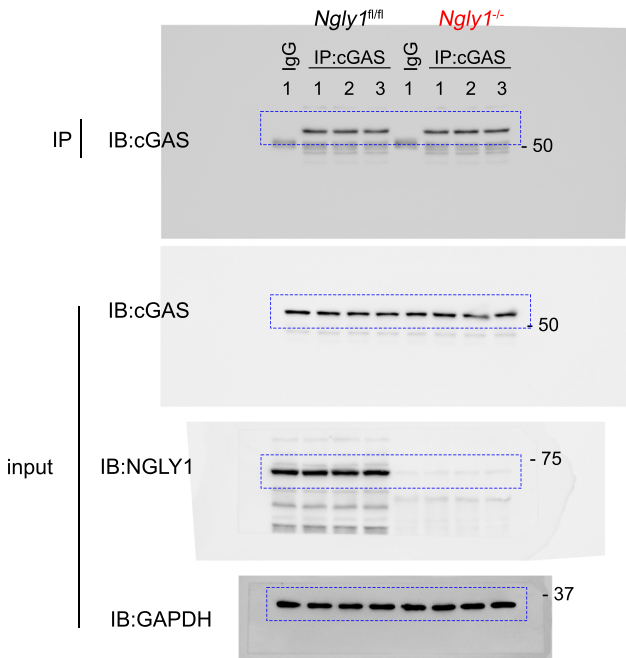

Supplement: SourceData FS5 — is the source file for Fig. S5. [file jem_20242296_sourcedatafs5.pdf]
